# Rabies Immunization Status of Dogs, Beijing, China

**DOI:** 10.3201/eid1706.101590

**Published:** 2011-06

**Authors:** Chao Wang, Ying Wang, Xiaoyan Du, Lin Zeng, Gang Dong, Yanhua Wu, Jing Lu, Deyou Wei, Xi Zhu, Guosheng Liu, Taiyun Zhao, Zhenwen Chen

**Affiliations:** Author affiliations: Capital Medical University, Beijing, People’s Republic of China (C. Wang, Y. Wang, X. Du, Y. Wu, J. Lu, Z. Chen);; Academy of Military Medical Sciences, Beijing (L. Zeng, T. Zhao);; Chinese People’s Liberation Army Center for Disease Control and Prevention, Beijing (G. Dong);; Animal Health Inspection Office of Fengtai District, Beijing (D. Wei);; Disease Control and Prevention Center of Fengtai District, Beijing (X. Zhu, G. Liu)

**Keywords:** rabies, antibody, titer, dog, survey, viruses, immunization, China, letter

**To the Editor:** In the People’s Republic of China, >3,000 persons die of rabies each year; most were infected by dog bites (*1*). Since 2000, the dog population in Beijing has increased dramatically, and the exact vaccination coverage and immunization status of dogs are not known.

During 2006–2009, to assist with governmental rabies control, Fengtai District was selected as a geographically representative area in Beijing in which to conduct a survey of rabies antibody titers in domestic dogs. Blood samples were randomly collected from 4,775 dogs in Fengtai District, which account for 3% of all registered dogs in the district. Rabies virus neutralization antibody (VNA) titers were detected by fluorescent antibody virus neutralization (*2*). In brief, VNA titers >0.5 IU indicated positive immunization, implying that the dog had an adequate level of antibody, and VNA <0.5 IU indicated negative immunization (*3*). The data were analyzed by 2-tailed χ^2^ test; p<0.05 was considered significant. Vaccination coverage and antibody levels were categorized either by dog’s function (guard or pet) or residence (urban or suburban) (Figure).

**Figure:**
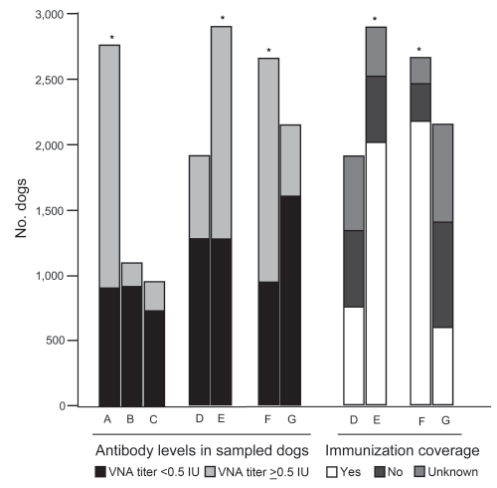
Rabies immunization status of dogs, Fengtai, Beijing, China. Immunization status and vaccination coverage were categorized according to dog vaccination background and rabies antibody level in each dog. A) Vaccinated, B) never vaccinated, C) unclear vaccination history; D) guard dog, E) pet dog; F) in urban areas, G) in suburban areas. *Significant difference (p<0.05) for dogs with positive antibody levels between A, B, and C; between D and E; or between F and G; or a significant difference (p<0.05) in dog immunization coverage between D and E or between F and G.

Most dogs with a history of vaccination were positively immunized (68.1%) (Figure, bar A), compared with 16.4% in the unvaccinated group (Figure, bar B), demonstrating that compulsory immunization is crucial to rabies control (*4*). Of 944 dogs with unclear vaccination history, 221 (23.4%) (Figure, bar C) had adequate antibody levels, possibly from undocumented vaccination or contact with rabies hosts. However, for 2006, 2007, 2008, and 2009, immunization coverage in the district was 55.0%, 53.8%, 67.4%, and 54.4%, respectively, all below the >70% criterion recommended by the World Health Organization (*5*). The results imply that much work still needs to be done by the Beijing government, not only to meet the World Health Organization immunization baseline but also to keep risk for a rabies epidemic in Beijing low.

Immunization coverage ratios differed significantly (p<0.05) between guard (39.3%) and pet dogs (69.5%) (Figure, bars D, E) and between urban (81.7%) and suburban areas (27.6%) (Figure, bars F, G). Consequently, the number of negatively immunized guard dogs was 1.68× lower than that for pet dogs (Figure, bars D, E) (p<0.05), and the number of positively immunized dogs in urban areas was 2.5× higher than that in suburban areas (Figure, bars F, G) (p<0.05).

In Beijing, guard dogs are usually raised by villagers to protect the house, whereas pet dogs are usually raised by city dwellers who treat dogs as friends. As a result, in urban areas dogs are registered and vaccinated in a timely manner by authorized pet hospitals (*6*). In suburban areas, however, dog management is deficient. For example, guard dogs in suburban areas are sometimes not vaccinated because the owner or veterinarian cannot safely restrain the dog for vaccination.

According to our study, >10% of unregistered dogs with no clear history of vaccination are not vaccinated during yearly vaccination programs. In Beijing during 2007–2009, of 9 cases of rabies in humans, 6 were associated with stray dogs (*7*), and most stray dogs were found in suburban areas. Hence, strategies to either reduce stray dogs in the city or to get such dogs under official management (e.g., include stray dogs in compulsory annual vaccination programs) are urgently needed.

In our opinion, policies related to dog registration, vaccination recording, and vaccination strategies need improvement in Beijing, especially in suburban areas. Although our report only focused on the Fengtai District, the findings could be helpful for the Beijing government for establishing strategies to control the rabies epidemic in the entire city.

## References

[1] Zhang YZ, Xiong CL, Xiao DL, Jiang RJ, Wang ZX, Zhang LZ, Human rabies in China. Emerg Infect Dis. 2005;11:1983–4. 16485502 10.3201/eid1112.040775 PMC3367615

[2] Yadav SC, Saini M, Raina OK, Nambi PA, Jadav K, Sriveny D. *Fasciola gigantica* cathepsin-L cysteine proteinase in the detection of early experimental fasciolosis in ruminants. Parasitol Res. 2005;97:527–34. 10.1007/s00436-005-1466-8 16222528

[3] Rokni MB, Massoud J, Hanilo A. Comparison of adult somatic and cysteine proteinase antigens of *Fasciola gigantica* in enzyme linked immunosorbent assay for serodiagnosis of human fasciolosis. Acta Trop. 2003;88:69–75. 10.1016/S0001-706X(03)00175-X 12943979

[4] Si H, Guo ZM, Hao YT, Liu YG, Zhang DM, Rao SQ, Rabies trend in China (1990–2007) and post-exposure prophylaxis in the Guangdong Province. [**PMID: 18717989**]. BMC Infect Dis. 2008;8:113. 10.1186/1471-2334-8-113 18717989 PMC2532688

[5] Coleman PG, Dye C. Immunization coverage required to prevent outbreaks of dog rabies. Vaccine. 1996;14:185–6. 10.1016/0264-410X(95)00197-9 8920697

[6] Wang Q, Wang Y, Wang D. An analysis of the rabies epidemic among clinic people exposed to rabies between 2001 and 2007 [in Chinese]. Can J Public Health. 2009;3:22–4.

[7] Tang Y, Yu L. How to effectively control the total number of out-of-control growth of domestic dogs in Beijing [in Chinese]. J Beijing People’s Police College. 2009;5:38–40.

